# Language Augmented Prediction

**DOI:** 10.3389/fpsyg.2012.00422

**Published:** 2012-10-29

**Authors:** Gary Lupyan

**Affiliations:** ^1^University of Wisconsin MadisonMadison, WI, USA

Clark's (in press) article makes a strong argument that prediction or reduction of “surprisal” comprises a synthesizing principle in understanding neural mechanisms. But if brains – all brains – are “essentially prediction machines,” how do we account for the apparently qualitative differences between humans and non-human animals in the ability to inspect and reflect on one's mental states, and to effectively foresee the consequences of various actions? For example, Spelke (2003) points out that although all animals find and recognize food, only humans developed the art and science of cooking. Although all animals have to understand (and predict!) the material world, only humans systematize their knowledge as science (p. 277). But we do not need to go into something as complex as formalized science to see the wide gap between human and non-human minds.

Imagine the simple task of pointing to a red box to get a reward, while ignoring the blue box. We can think of success as the mapping between the sensory input and the motor output that minimizes surprisal. Many animals can succeed on this task after being trained – their behavior nudged gradually by rewards until the generated predictions match the contingencies of the task. In contrast, humans can succeed without any training at all, simply by being told what to do!^1^ We often take this ability for granted, but without it, all human learning would require direct experience with the domain (e.g., see Carvalho et al., 2008 for an account of the laborious trial-and-error learning in tool-using chimpanzees). If all brains are surprisal-reducing machines, what is it about human brains that allows them to be guided so effectively, often foregoing laborious trial-and-error tweaking?

A common solution is to posit that humans evolved a special neural mechanism for re-representing information in a way which allows complex inferences, cognitive flexibility, language, (and self-awareness itself; Penn et al., 2008). The solution Clark offers – cursorily in the target article (§3.4, note xxxii) and more in depth in earlier work (e.g., Clark, 1998) – is that language together with other aspects of symbolic culture augment an otherwise un-remarkable pattern-completion, surprisal-reducing brain with faculties we have come to uniquely associate with the human mind, e.g.:

“…linguistic formulation makes complex thoughts available to processes of mental attention. [It] enables us, for example, to pick out different elements of complex thoughts and to scrutinize each in turn” (Clark, 1998, p. 177–198).

Clark writes that “symbol-mediated loops” can “enable new forms of reentrant processing”(§3.4), but how does this work? Putting aside the question of how symbolic language and culture evolve in the first place, how might an agent's experience with symbols augment the prediction machinery? Answers to this question have tended to focus on (1) agent-level uses of language: explicit linguistic strategies such as verbal rehearsal, and mnemonic and chunking strategies (e.g., remembering an arbitrary sequence of letters by thinking of a sentence containing words that begin with those letters, or learning to tie a knot by thinking of a rabbit going in and out of a hole), and (2) explicit verbal mediation, i.e., “thinking in words.” Indeed, this introspection of thinking in words is often so strong that it leads researchers to conflate that feeling of talking to oneself with the format of conceptual representations (Ryle, 1968; e.g., Carruthers, 2002; Levinson, 1997 for discussion).

This confusion can be clarified by considering the role language can play in generating top-down predictions (Lupyan, 2012a,b for discussion). A growing body of work suggests that language interfaces directly with the surprisal-reducing machinery at the core of predictive-coding models. Consider a task in which one hears an auditory cue (e.g., a barking sound) and then sees a picture (e.g., a dog). The goal is to respond “yes” if the cue and picture match at a conceptual level, and “no” otherwise (e.g., a car following a barking sound). The better the match between the top-down predictive signal and the bottom-up activation produced by the probe, the faster (or more accurately) subjects can respond. Lupyan and Thompson-Schill (2012) found that linguistic cues (“dog”) were more effective than non-linguistic cues (e.g., a barking sound, a car horn), even though both cue types were judged as equally predictive and unambiguous of the associated category. As the delay between the cue and probe was increased, the difference between the verbal and non-verbal-cue conditions also increased. Under the influence of the label (through hypothesized top-down effects), the resultant representations appeared to become more similar across subjects with increasing delays in a way that they did not on trials without the verbal label. This provides a basic demonstration of how verbal labels act as “cues” (Elman, 2009) altering how knowledge (e.g., of what a dog looks like) is brought online.

This effect of labels as “cues” – augmenting the processing of incoming sensory information – can also be observed in simple visual discrimination and even simple detection tasks (e.g., Lupyan, 2008; Lupyan and Spivey, 2010). Words appear to serve as especially efficient *category* cues to the system, selectively activating the features most typical/diagnostic of the target category, resulting in representations that allow more efficient discrimination between the target and non-target stimuli or between signal and noise (Ward and Lupyan, 2011). Indeed it is this that may be responsible for the facilitatory role labels appear to play in the learning of some novel categories (Lupyan et al., 2007; see Lupyan, 2012a for a computational model).

This approach of up- or down-regulating language can be used to partially overcome the limitation of not having access to human brains unaided by language^2^. Even small linguistic tweaks can augment ongoing processing even in apparently low-level perceptual tasks. By considering the functions language has on the predictive mechanisms of the brain, we can gain further insights not just in domains where language acts as a tool – allowing us to do such things as guide behavior by writing down cooking recipes – but on such a fundamental question as how it is that humans can tell each other what to do!

## Footnotes

^1^It is tempting to assume that the inability to tell other animals what to do is a problem of communication. But the problem here is not communication, but that of flexibly activating task-relevant mappings. Language is not necessary for communicating instructions for a task as simple as this, but experience with language *may* be necessary for the ability to flexibly deploy task-relevant stimulus-response mappings without environmental guidance.

^2^Special cases such as deaf children not exposed to Sign can be informative, but what is typically ignored is that such individuals nevertheless interact with linguistic beings and live in a world shaped by language and symbolic culture.
